# Can Soybean Cultivars with Larger Seed Size Produce More Protein, Lipids, and Seed Yield? A Meta-Analysis

**DOI:** 10.3390/foods11244059

**Published:** 2022-12-15

**Authors:** Cailong Xu, Tingting Wu, Shan Yuan, Shi Sun, Tianfu Han, Wenwen Song, Cunxiang Wu

**Affiliations:** Institute of Crop Sciences, Chinese Academy of Agricultural Sciences, National Soybean Industrial Technology R & D Center, Beijing 100081, China

**Keywords:** soybean, seed size, seed yield, protein and lipids productivity

## Abstract

Increasing soybean production and ensuring greater access to soybean protein and lipids is critical for global food security and human health. Seed size (i.e., seed weight) is one of the most important agronomic traits of soybean, which not only determines the seed yield, but can also affect the yield of protein and lipids. In China, farmers favor soybean cultivars with large seeds, which they believe produce more protein and lipids; however, experimental evidence supporting this belief is lacking. Therefore, we conducted field experiments from 2017 to 2020 at 35 locations across the Huang-Huai-Hai region (HHH) of China with 64 soybean cultivars. The seed yield, seed protein content, and seed lipids content of soybean, and their relationship with seed size were investigated. The highest seed yield (i.e., seed weight per unit area) was 2996.5 kg ha^−1^ in the north of HHH. However, the highest seed protein content was found in the south of HHH (42.5%) for the higher temperature, which was significantly higher than that of the middle (41.7%) and north of HHH (40.2%). In contrast, the highest seed lipids content was 20.7% in the north of HHH. Temperature, which had a path coefficient on seed yield of 0.519, can promote soybean seed yield. The correlation analysis indicated that the selection of the large seed size cultivar did not increase seed yield, and even led to a reduction of seed yield under high-yield environmental conditions. The seed protein content of soybean was not increased in the cultivars with large seed sizes. In addition, under different levels of seed lipids content (<20.30% or >20.30%), a significantly negative relationship was found between seed lipids content and hundred seed weight. Therefore, it is recommended that farmers choose to plant cultivars with smaller soybean seed sizes, so as to ensure high and stable soybean seed yield and obtain more vegetable protein and lipids per unit area.

## 1. Introduction

With population growth, changing diet, and diminishing supplies of fossil fuels, the global demand for food and biofuel will continue to increase [1,2,3]. Soybean is an important source of vegetable lipids and protein, which account sequentially for 18%–20% and 35%–40% of seed dry weight, respectively [4,5]. Driven by the demand of consumers, the global cultivated area of soybean has increased from 77 million ha in 2001 to 127 million ha in 2020 [6]. In the future, soybean production will continue to increase in order to ensure the supply of high-quality vegetable protein and lipids worldwide.

China is the largest soybean consumer in the world, consuming more than 120 million tons a year, accounting for more than a third of the global soybean production [7]. The large demand for vegetable proteins and vegetable lipids has been the main driver behind the surge in soybean consumption in China [8]. Increasing soybean seed, soybean protein, and soybean lipids under limited arable land is important for the development of the soybean industry in China [9]. The soybean seed yield in China, which accounts for less than 60% of the yield in the US, has stagnated [10]. Increasing the seed yield of soybean may increase the yield of soybean protein and lipids [11]. However, previous studies have found variation in the soybean seed yield of the same cultivar in different years and environmental conditions during the same growing season [5,12]. The variations in seed yield were likely due to soil type, management practices, and climate conditions, especially the latter [10,13].

Previous studies have shown that climate was the most important source of variation for soybean protein and lipids content in seeds [14,15]. Under insufficient light conditions, soybean will respond with elongation of stem internode, which leads to lodging risk and disorder of the distribution of photosynthate, resulting in seed yield reduction and quality deterioration [16]. Under high temperatures, the number of cells in soybean cotyledons decreased significantly, and the filling capacity of dry matter was weakened, which resulted in slower seed weight gain and more shriveled seeds [17,18]. In addition, climatic factors, such as the duration of sun and rainfall, can also affect seed yield and seed composition [5,19]. However, many of the studies described above were investigated under controlled greenhouse and growth chamber conditions; therefore, it is essential to study the effect of climate on soybean seed yield in natural conditions.

In order to develop high-yield cultivation measures adapted to the climate in different regions of China, we further need to clarify the relationship between soybean seed yield and soybean seed quality of the cultivars grown in China. Soybean has a long history of cultivation of more than 5000 years in China, resulting in characteristics specific to soybean cultivars grown in the country [20,21]. The most prominent features of Chinese soybean cultivars are their large seeds, weighing 180–290 mg per seed [10], compared with 102–195 mg per seed for North American soybean cultivars [13,22,23,24]. The high seed weight of Chinese soybean cultivars stems from the belief of Chinese farmers that the bigger the seed, the higher the protein content. Thus the demand by farmers for a larger size seed has led to this trend in soybean breeding in China. However, experimental evidence supporting the belief that “bigger is better” is lacking.

In fact, we speculate that large seed cultivars may have poor tolerance to adverse environmental conditions, which increases variability in soybean production for two reasons. First, soybeans are dicotyledons, which during seed germination, the top hook is used to break the soil, driving the cotyledons to emerge [25]. Larger seeds experience more resistance when they emerge, which can damage cotyledons and apical meristems, affecting the emergence and survival rates of soybean [26]. Second, farmers often increase the seeding rate to ensure the survival of seedlings in the field. However, the increase in sowing quantity increases the input required to gain a seed yield. Further, the increase in seed weight results from plants continuously filling their seeds with dry matter [27,28], which is negatively impacted by unfavorable environmental conditions [29,30]. Therefore, cultivars with high seed weight may need to have continuously favorable environmental conditions during the filling period, otherwise, their seed weight will be reduced.

The objectives of this study were to (1) clarify the spatial performance of seed, protein, and lipid weight per area in soybean productivity in the Huang-Huai-Hai region (HHH) of China, and their response to meteorological conditions; (2) investigate the relationship between seed size and the stability of seed yield of soybean cultivar; and (3) analyze the relationship between seed size of soybean cultivar as well as their protein and lipids content. The findings from this study will provide insight into the relationship among soybean seed size, seed yield, and protein and lipids content of the seed, thus allowing for improved overall soybean industry in China.

## 2. Materials and Methods

### 2.1. Study Area

**Figure 1:**
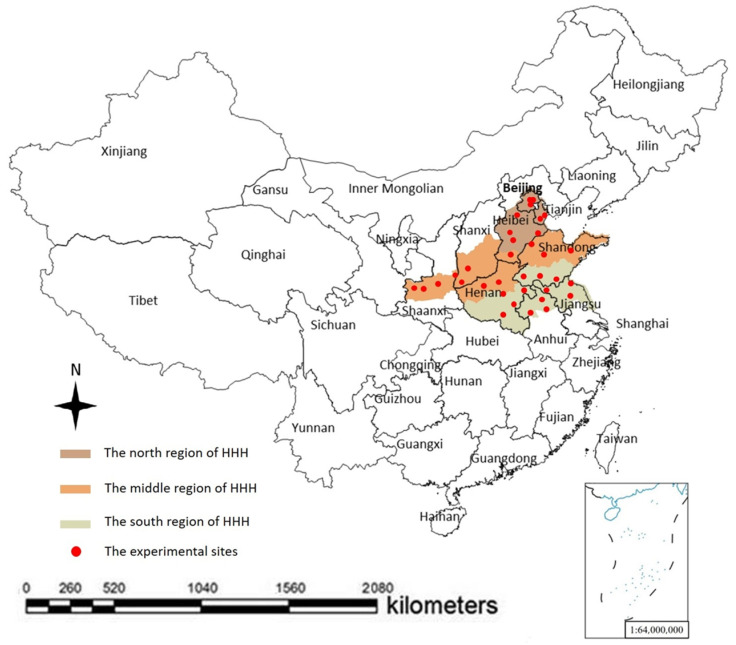
The spatial distribution of the experimental sites and soybean fields in Huang-Huai-Hai region, China.

Field trials were conducted in HHH, an area with a warm and semi-humid continental monsoon climate (Figure 1). The winter in HHH is cold and dry, while summer is hot and wet; the annual precipitation is extremely variable, ranging from 100 to 300 mm, of which 70–80% falls in Jun.-Aug. [10]. The main cropping system in HHH consists of two harvests of complementary crops per year, including wheat-maize or wheat-soybean. The summer crops are planted in early June, immediately after the winter wheat (*Triticum aestivum* L.) harvest, and then they are harvested at the beginning of October. The soybean planting area in HHH accounts for nearly 30% of the total soybean planting area in China and is considered a high-quality soybean-producing area due to the high protein content of the harvest [10].

### 2.2. Field Experiment

Field trials were conducted in 35 locations across the HHH from 2017 to 2020. The 35 locations were divided into three major subregions based on differences in geographic regions, climate, water resources, and the growth period of soybean [31]: including 10 locations in the north, 12 in the middle, and 13 in the south of HHH (Figure 1, Table S1). In this study, the soybean cultivars suitable to each subregion were used, including those that have been released and those that will be released (Table S1). At each test location, the experiments were conducted using a randomized complete block design with three replicates, and each included the soybean cultivars (Table S1). Each plot was 18 m^2^ (3 m × 6 m). Soybean seeds were sown at a row spacing of 0.4 m in both years. Soybean was planted by hand from 10 June to 20 June with a planting density of 19–23 × 10^4^ p ha^−1^, and harvested from 20 September to 10 October. Fertilizers were applied at rates of 25–38 kg N ha^−1^, 35–52 kg P_2_O_5_ ha^−1^, and 25–38 kg K_2_O ha^−1^ in the north, middle, and south subregions, respectively. The amount of fertilizer applied in each subregion varied to be optimal for different climates. Irrigation was applied as required in each experimental site during the growth period of soybeans. Weeds and insect pests were managed well at each site.

### 2.3. Data Collection

After physiological maturity (R7), all soybean plants in the central four rows of each plot, representing an area of 9.6 m^2^ (1.6 m × 6 m), were harvested by hand to measure seed yield (seed weight per unit area, kg ha^−1^). Seed yield and hundred seed weight were reported at a moisture content of 13.0%. The hundred seed weight was the mean of three random samples of 100 seeds. Seed yield stability index (YSI) was calculated using the following equation as described in previous studies [12]: YSI = (Y_mean_ – SD)/Y_max_, where, Y_mean_, SD, and Y_max_ are the mean, standard deviation, and the maximum of soybean seed yield in the HHH region across all cultivars and planting years. 

The soybean seeds of each cultivar were ground and passed through a 60-mesh filter for quality trait analysis. The seed protein contents of different soybean cultivars were analyzed using the semi-micro-Kjeldahl method according to Xu et al. [32]. Protein yield (protein weight per unit area, kg ha^−1^) was the product of seed protein content multiplied by soybean seed yield. The seed lipids content was determined using the Soxhlet extraction method according to Song et al. [5]. Lipids yield (lipids weight per unit area, kg ha^−1^) was the product of the seed lipids content multiplied by soybean seed yield. 

The climate data were recorded at each experimental site during the study years from the National Meteorological Information Center (http://data.cma.cn accessed on 1 January 2021) of the China Meteorological Administration. The accumulated temperature (≥10 °C), total precipitation, average temperature, sunshine hours, and diurnal temperature range (DTR) from sowing to maturity at each site during the study were averaged across all cultivars and planting years. The DTR was the range of the daily maximum and minimum temperatures.

### 2.4. Statistical Analyses

The data were tested for normal distribution using the Shapiro-Wilk method in SPSS 25. Statistically significant differences for seed yield, hundred seed weight, seed protein content, seed lipids content, protein yield, and lipids yield in different regions were computed using one-way ANOVA at the 0.05 level of probability conducted in SPSS 25. A path analysis of the relationship between soybean seed yield and meteorological parameters was calculated by the standard regression coefficient method using SPSS 25.

## 3. Results

### 3.1. Seed Yield, Hundred Seed Weight, and Protein and Lipids Content of Seed

**Figure 2:**
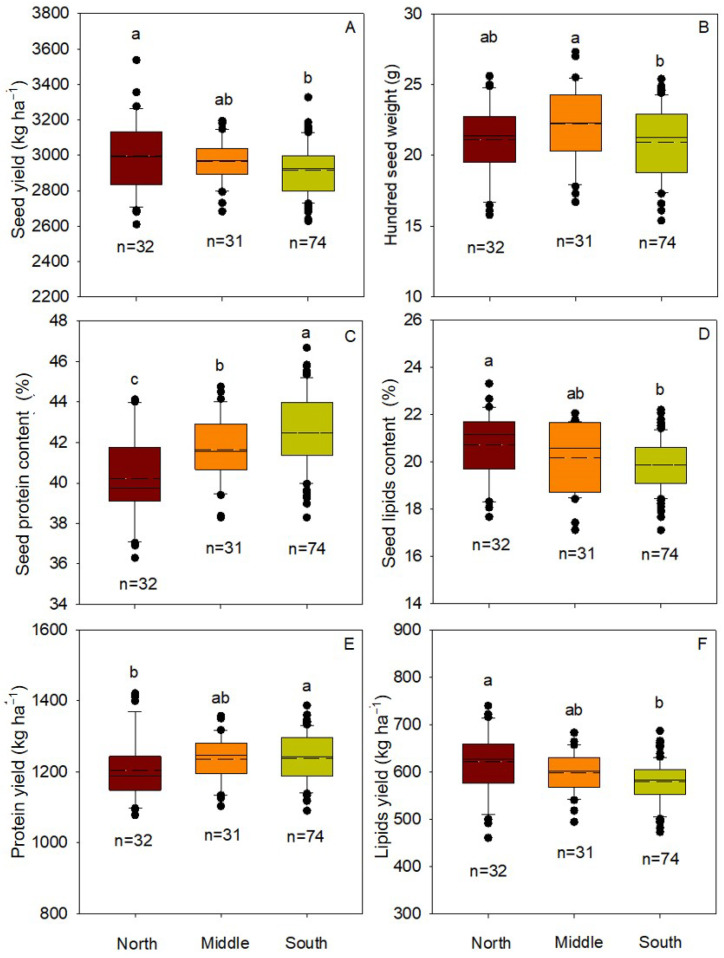
Seed yield (**A**), hundred seed weight (**B**), seed protein content (**C**), seed lipids content (**D**), protein yield (**E**), and lipids yield (**F**) of soybean across the Huang-Huai-Hai region of China. The same lowercase letters for the different regions indicate that the differences are not statistically significant at *p* < 0.05. The solid and dashed lines within the boxes indicate medians and means, respectively; the upper and lower edges of the box represent the 25th and 75th percentiles of all the data, and the bottom and top bars represent the 5th and 95th percentiles, respectively. Solid black circles are outliers.

Among the three soybean-growing regions, the seed yield was the highest in the north, followed by the middle, with the lowest seed yield in the south (Figure 2 and Figure S1). The highest average seed yield was 2996.5 kg ha^−1^ (range: 2608.5–3537.0 kg ha^−1^) in the north, which was significantly (*p* < 0.05) greater than the lowest average seed yield of 2916.1 kg ha^−1^ (range: 2626.5–3327.0 kg ha^−1^) in the south (Figure 2A). The highest average hundred seed weight was 22.3 g (range: 16.7–27.3 g) in the middle of HHH, which was 6.7% higher than that in the south, which had the lowest hundred seed weight (Figure 2B). However, the highest average seed protein content was found in the south of HHH (42.5%, range: 38.3%–46.7%), which was significantly (*p* < 0.05) higher than that of the middle (41.7%, range: 38.3%–44.8%) and the north (40.2%, range: 36.3%–44.1%) by 1.9% and 5.7%, respectively (Figure 2C). The highest average seed lipids content was 20.7% (range: 17.7%–23.3%) in the north, which was significantly (*p* < 0.05) higher than the lowest average seed lipids content of 19.9% (range: 17.1%–22.2%) in the south (Figure 2D). In general, soybean protein yield in the HHH was lower in the north and higher in the south, while the average lipids yield was higher in the north and lower in the south (Figure 2E,F). 

### 3.2. Differences of Meteorological Factors and Their Relationship with Seed Yield

**Figure 3:**
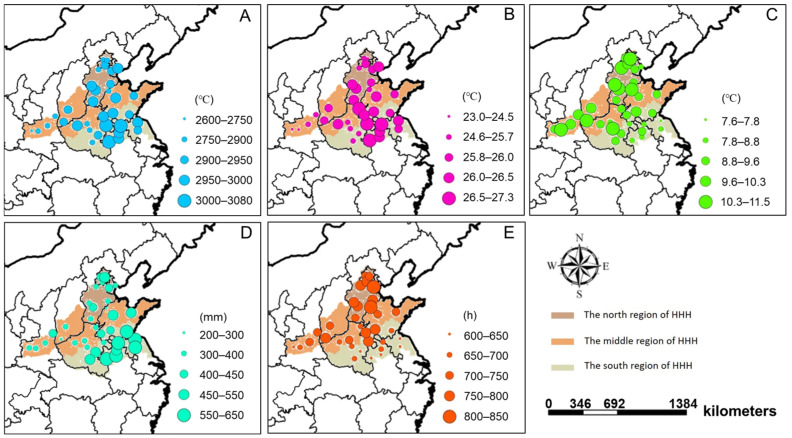
Spatial distributions of temperature accumulation (≥10 °C, **A**), average temperature (**B**), diurnal temperature range (**C**), total precipitation (**D**), and sunshine hours (**E**) from sowing to maturity during the study years.

**Figure 4:**
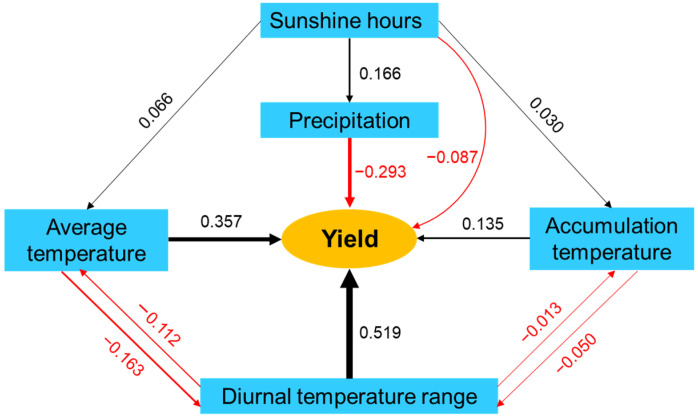
Path analysis of the relationships among seed yield, sunshine hours, total precipitation, average temperature, temperature accumulation (≥10 °C), and diurnal temperature range. Arrows point from causes and effects. Black arrows represent positive effects, and red arrows represent negative effects. The values along the arrows are path coefficients.

In HHH, the accumulated temperature (≥10 °C) varied from 2596.9 to 3085.1 °C across years and sites, with an average of 2926.6 °C, which was generally lower in the north and higher in the south (Figure 3A). The accumulated temperature (≥10 °C) in the north and middle regions were 2913.7 °C (range: 2766.8–2987.4) and 2900.7 °C (range: 2596.9–3085.1 °C), respectively, while it was 2960.5 °C (range: 2894.0–3038.7 °C) in the south. The average temperature in HHH ranged from 23.0 to 27.3 °C across years and sites, with an average temperature of 25.9 °C. As above, the average temperature was lower in the north and higher in the south (Figure 3B). The average temperature in the north and middle subregions was 25.8 (range: 24.5–26.4 °C) and 25.7 °C (range: 23.0–27.3 °C), respectively, while it was 26.2 °C (range: 25.6–26.9 °C) in the south. Compared with accumulated temperature (≥10 °C) and average temperature, the variation in the diurnal temperature range was dramatic in HHH. The diurnal temperature range varied from 7.6 to 11.5 °C with an average of 9.5 °C. The diurnal temperature range decreased from north to south (Figure 3C). The highest diurnal temperature was 10.0 °C (range: 7.9–11.5 °C) in the north subregion, followed by middle 9.6 °C (range: 8.6–10.8 °C), and south 8.9 °C (range: 7.6–10.4 °C). As indicated by the path coefficient on seed yield of 0.519, the temperature can promote soybean seed yield formation, especially in the diurnal temperature range (Figure 4).

Precipitation in HHH was another meteorological indicator with large spatial variability (Figure 3D). The precipitation varied greatly from 197.3 to 628.1 mm, with an average of 434.1 mm. The precipitation was concentrated in the south subregion (range: 408.0–628.1 mm; average = 524.7 mm). While the precipitation in the north and middle subregions were 388.8 (range: 281.5–490.2) and 384.7 mm (range: 197.3–535.0 mm), respectively. The path coefficient on seed yield was −0.293, indicating that precipitation inhibited soybean seed yield (Figure 4). 

The sunshine hours also varied among subregions in HHH. Sunshine hours varied from 603.4 to 859.5 h, with an average of 717.9 h. Sunshine hours decreased from north to south (Figure 3E). The highest and lowest sunshine hours were 782.7 h (range: 736.0–859.5 h) in the north subregion and 675.5 h (range: 603.4–780.7 h) in the south, respectively. The central sites had an average of 710.2 h sunshine hours (range: 627.5–751.8 h), which was the median in HHH. There was a weakly negative correlation between sunshine hours and soybean seed yield; the path coefficient of sunshine hours on seed yield was −0.087 (Figure 4).

### 3.3. Relationship of Seed Yield Stability and Properties of Seed Quality with Seed Size 

**Figure 5:**
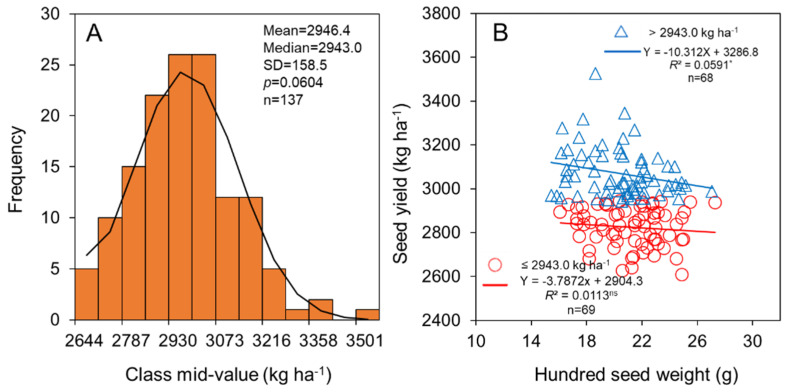
Relationship of seed yield (**A**) and hundred seed weight (**B**) for soybean. * indicates significance different at the 0.05 probability levels, while ns indicates not significant different.

**Figure 6:**
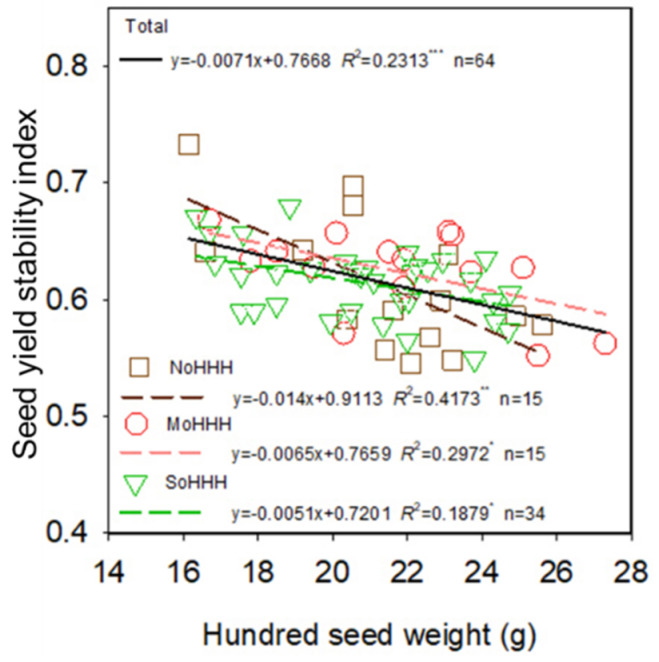
Relationship of the seed yield stability index and hundred seed weight for soybean. *, **, and *** indicate significance different at the 0.05, 0.01, and 0.001 probability levels, respectively.

The soybean seed yield in HHH ranged from 2608.5 to 3537.0 kg ha^−1^, with mean and median values of 2946.6 and 2943.0 kg ha^−1^, respectively (Figure 5A). Correlation analyses highlighted different relationships between soybean seed yield and hundred seed weight under different seed yield grades. Soybean seed yield was significantly (*p* < 0.05) negatively correlated with hundred seed weight for seed yield levels of >2943.0 kg ha^−1^, while no significant (*p* > 0.05) relationship was found between seed yield and hundred seed weight under seed yield levels of <2943.0 kg ha^−1^ (Figure 5B). This finding suggests that selection of the large-size seed cultivar did not contribute to increasing soybean seed yield, and even led to soybean seed yield reduction in high-yield environments. The seed yield stability index was significantly (*p* < 0.001) negatively correlated with hundred seed weight, which further confirmed the above finding (Figure 6).

**Figure 7:**
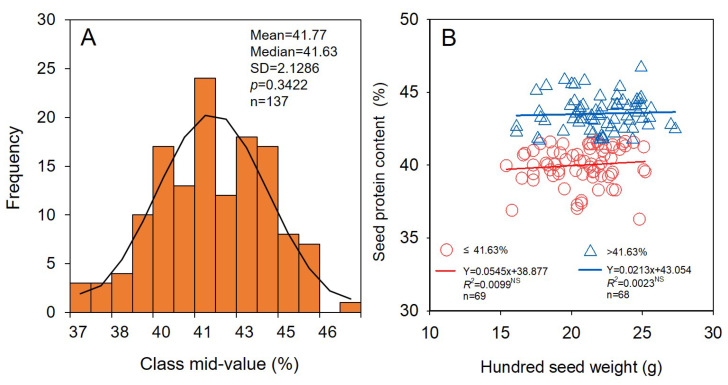
Relationship of seed protein content (**A**) and hundred seed weight (**B**) for soybean. ns indicates not significant different at the 0.05 probability levels.

The relationships of seed protein content, seed lipids content, seed yield, and seed yield stability index with hundred seed weight of soybean were investigated. The seed protein content of soybean in HHH ranged from 36.3% to 46.7%, with mean and median values of 41.77% and 41.63%, respectively (Figure 7A). No significant (*p* > 0.05) differences were found between seed protein content and hundred seed weight under any seed protein content grades (<41.63% or >41.63%) of soybean (Figure 7B). This suggests that the seed protein content of soybean was not increased with large seed sizes of cultivars in HHH. 

**Figure 8:**
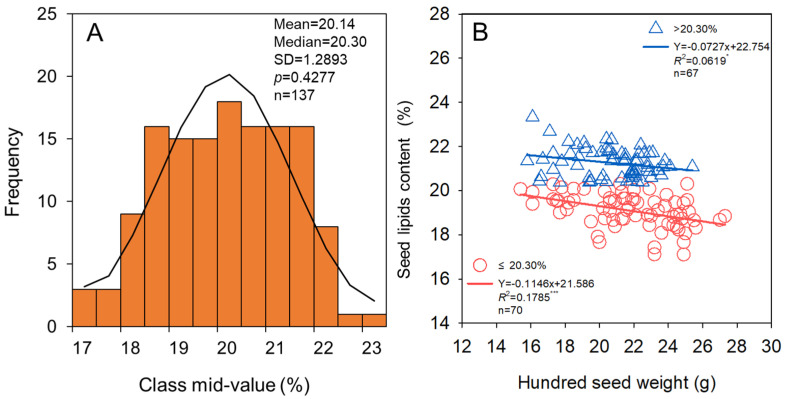
Relationship of seed lipids content (**A**) and hundred seed weight (**B**) for soybean. * and *** indicate significance different at the 0.05 and 0.001 probability levels, respectively.

The seed lipids content of soybean in HHH ranged from 17.1% to 23.3%, with mean and median values of 20.14% and 20.30%, respectively (Figure 8A). In addition, under different seed lipids content levels (<20.30% or >20.30%), significant (*p* < 0.001 and *p* < 0.05, respectively) negative relationships were both found between seed lipids content and hundred seed weight of soybean (Figure 8B). Thus, the pursuit of soybean cultivars with large size cultivars would lead to the loss of seed lipids content.

## 4. Discussion

### 4.1. Spatial Characteristics of Soybean Production and Their Relationship with Climatic Factors in HHH 

To achieve high yield and efficient utilization of resources for chosen cultivars, the crop cultivar must be carefully selected to match regional environmental conditions [10,33], especially for crops such as soybeans, which are particularly sensitive to photoperiod and temperature [17,34,35]. The HHH region spans nearly 10 latitude lines (32.9–40.2° N) from north to south and has diverse topography and climate. In the present study, seed yield, seed lipids content, and lipids yield were highest in the north, followed by the middle, which was the lowest in the south across the HHH region; the seed protein content and protein yield showed the opposite trend (Figure 2). Spatial differences in the meteorological data were also found across the HHH. The accumulated temperature and average temperature were generally low in the north and high in the south, while the diurnal temperature range and sunshine hours showed different spatial distributions (Figure 3). Precipitation was greater in the southern subregion compared to the northern subregion (Figure 3). Previous studies showed that the variation in soybean productivity in different regions is due to the differential distribution of meteorological factors among different regions [5,36]. 

The soybean seed yield was found to be significantly and positively correlated with temperature, especially the diurnal temperature range (Figure 4). Temperature largely controls the physiological metabolism of crops, which determines growth and seed yield [37,38]. Isoda et al. [39] reported that the Japanese soybean cultivar Toyokomachi achieved 9200 kg ha^−1^ due to the abundant luminous radiation and larger temperature difference between day and night in Xinjiang Province, especially the latter. In addition, a higher diurnal temperature range increased seed lipids content and decreased seed protein content in the present study, which was consistent with the previous results [5]. An increase in the rate of respiration at night, mainly caused by high night temperatures, can limit carbon sequestration and the translocation of C and N to grains [40,41]. Sunshine hours also play a role in the amount of accumulated solar energy, which promotes the accumulation of biomass by crops [33,42,43]. However, a weakly negative correlation was found between soybean seed yield and sunshine hours in the present study (Figure 4). This result may be related to the fact that soybean is a short-day crop [44]. Long-day conditions can easily lead to the failure of soybean plants to flower and seed normally, resulting in a decline in seed yield.

### 4.2. Soybean Cultivars with Smaller Seed Size Have Stronger Seed Yield Stability

Obtaining a continuous and stable high seed yield to ensure food security and high nutrition is the ultimate goal of agricultural production [3,45]. In agricultural production, crop yield is affected by cultivar, climate, soil, pests and diseases, field management, and many other factors [46,47]. Multi-year and multi-site experiments are necessary to verify the stability of crop yield potential and the potential for the productivity of an environment [44,48]. In the present study, we evaluated the potential for the productivity of the environment of each subregion of the HHH on the basis of a multi-year, multi-site, and multi-cultivar experiment (Figure 1 and Table S1). Our findings provide a reference for optimizing soybean production and improving soybean seed yield. However, yield gaps between modeled yield and farmers’ yields were existent in a productivity system, which needs to be closed by selecting improved cultivars and optimizing cultivation practices [49]. Improving soybean seed yield and its seed yield stability (Figure 6) is an important measure to close the regional yield gap and it is also the focus of this study.

Seed size is one of the primary indexes of soybean seed yield [50]. In the present study, planting the soybean cultivar with a large seed size was not beneficial to the final seed yield, especially under high-yield conditions (Figure 5). In addition, a negative correlation was found between the seed yield stability index and hundred seed weight, indicating that the soybean cultivar with a larger seed size could not ensure a continuous and stable high seed yield (Figure 6). Seed weight was positively correlated with grain filling rate and grain filling duration, which had been verified in several studies about maize, rice, soybean, and so on [32,51,52]. Thus, cultivars with larger seed weights require continuous absorption of biomass over an extended period of time to fill the seed reservoir [28,29,30]. However, external factors, such as weather, climate, insect pests, and field management practices, often affect dry matter accumulation, which in turn inhibits seed filling, resulting in low seed weight [53]. These results indicated that compared with small-seed cultivars, large-seed cultivars were more susceptible to external influences during seed filling, resulting in fewer full seeds and lower seed yield. Therefore, the selection of smaller seed soybean cultivars is more conducive to high and stable seed yield.

### 4.3. Soybean Cultivars with Larger Seed Size Can Not Produce More Protein and Lipids 

Compared with that of other major crops, such as rice, wheat, and maize, the main purpose of soybean cultivation is to yield more vegetable protein and lipids [5,54]. Increasing seed protein and lipids content is an important way to obtain more protein and oil [55,56]. Plant breeders are also working to produce high-yielding and high-quality soybean cultivars [57]. We found that the soybean seed protein content did not change significantly with the increase in seed weight (Figure 7). These results were different from previous studies, which showed that the smaller the soybean seed, the higher the protein content [57]. The reason for the contrasting results may be that Wang et al. [57] primarily focused on wild soybeans, while our study was conducted on cultivated soybeans. Chinese breeders have fully considered the synergistic increase in seed yield and seed protein content when selecting soybean cultivar [56]. In the present study, we concluded that growers can obtain more soybean protein by increasing soybean seed yield rather than the seed size. 

**Figure 9:**
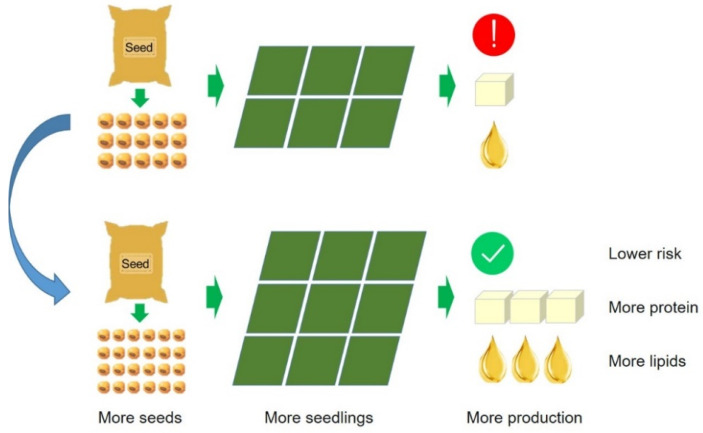
Soybean cultivars with smaller seed sizes can produce more protein and lipids.

The seed lipids content decreased with the increase in seed weight in the present study (Figure 8), which was consistent with previous studies conducted by Song et al. [58] and Assefa et al. [58]. Other studies have shown a significant negative correlation between protein content and lipids content in seeds [59,60,61]. This is likely because pyruvate is required for both fat synthesis and protein synthesis, and there is competition between the two processes for synthetic substrates [62,63]. Therefore, with the increase in seed weight, the stable synthesis of protein in seeds inhibited the formation of lipids, resulting in the reduction of lipids content in seeds. Our results provide important insights into seed size selection for soybean production, which not only reduces the seed input but also helps stabilize and improve soybean protein, lipids, and seed yield (Figure 9).

## 5. Conclusions

In the present study, the average seed yield, seed lipids content, and lipids yield of soybean in HHH were 2946.4 kg ha^−1^, 20.14%, and 593.8 kg ha^−1^, respectively, and was highest in the north and lowest in the south. The average seed protein content and average protein yield were 41.77% and 1229.5 kg ha^−1^, respectively, which had an opposite trend compared with the average seed yield, seed lipids content, and lipids yield. The diurnal temperature range was responsible for these differences. In addition, higher seed yield, stronger seed yield stability, and higher seed lipids content were found in the soybean cultivars that have smaller seed sizes. We confirmed that the seed protein content of soybean did not decrease with the decrease in seed size. Therefore, it is recommended that farmers plant soybean cultivars with smaller seed sizes, which should ensure a high and stable seed yield and allow for a greater vegetable protein and vegetable oil harvest.

## Data Availability

The data presented in this study are available on request from the corresponding author.

## Supplementary Materials

The following supporting information can be downloaded at: https://www.mdpi.com/article/10.3390/foods11244059/s1, Figure S1: Cumulative probability of seed yield (A), hundred seed weight (B), seed protein content (C), seed lipids content (D), protein yield (E), and lipids yield (F) of soybeans across the Huang-Huai-Hai region, China; Table S1: Details of experimental sites and varieties.

## Abbreviations

HHH	Huang-Huai-Hai region
DTR	diurnal temperature range
YSI	seed yield stability index
