# Supplementary material for: Can Soybean Cultivars with Larger Seed Size Produce More Protein, Lipids, and Seed Yield? A Meta-Analysis
Source: Foods. 2022 Dec 15;11(24):4059. doi: 10.3390/foods11244059 (PMC9777928; doi:10.3390/foods11244059)
Supplement: Supplementary file 1 [file foods-11-04059-s001.zip › foods-2048984-supplementary.pdf]

Table S1. Details of experimental sites and varieties.

Soybean-Growing Region in HHH	Experiment Site (°N, °E)	Variety (Growing Season)
North	**Beijing:** Shunyi District (40.1, 116.7), Changping District (40.2, 116.2), Daxing District (39.7, 116.3). **Tianjin:** Ninghe District (39.3, 117.8), Xiqing District (39.1, 117.0). **Hebei Province:** Yixian County (39.4, 115.5), Shijiazhuang City (38.0, 114.5), Nanpi County (38.0, 116.2), Ningjin County (37.6, 114.9). **Shandong Province:** Dezhou City (37.4, 116.4)	Jidou12 (2017-2020), Ji1507 (2017-2018), Jidou23 (2018-2019), Andou1498 (2018-2019), Kedou13 (2018-2019), Shidou17 (2018-2019), Zhonghuang204 (2018-2019), Zhonghuang206 (2018-2019), Zhonghuang605 (2018-2019), Zhonghuang80 (2018-2019), Handou20 (2019-2020), HN0811 (2019-2020), Jidou32 (2019-2020), Zhonghuang205 (2019-2020), Zhonghaung313 (2019-2020)
Middle	**Hebei Province:** Handan City (36.6, 114.5). **Shandong Province:** Weifang City (36.7, 119.2), Jinan City (36.7, 117.1). **Henan Province:** Puyang City (35.8, 115.0), Zhengzhou City (34.7, 113.6), Luoyang City (34.6, 112.5). **Shanxi Province:** Linfen City (36.1, 111.6), Yuncheng City (35.0, 110.1). **Shaanxi Province:** Huayin City (35.6, 110.1), Fuping County (34.8, 109.2), Baoji City (34.4, 107.4), Yangling District (34.3, 108.1)	Handou5 (2017-2020), Hedou23 (2017-2020), Han13-99 (2018-2019), Jindou50 (2018-2019), Luo1304 (2018-2019), Pudou561 (2018-2019), Qihaung39 (2018-2019), Shanning30 (2018-2019), Shengdou8 (2018-2019), Shi936 (2018-2019), Wansu1015 (2018-2019), Xiangfeng3 (2018-2019), Zhonghuang207 (2018-2019), Zhonghuang311 (2018-2019), Qihuang34 (2019-2020)
South	**Shandong Province:** Jining City (35.4, 116.6), Heze City (35.2, 115.5), Linyi City (35.1, 118.4). **Henan Province:** Shangqiu City (34.4, 115.7), Xuchang City (34.1, 113.8), Zhoukou City (33.6, 114.7), Zhumadian City (33.0, 114.0). **Anhui Province:** Suzhou City (33.6, 117.0), Longkang Town (33.1, 116.9), Fuyang City (32.9, 115.8). **Jiangsu Province:** Guanyun County (34.3, 119.2), Xuzhou CIty (34.2, 117.3), Huaian City (33.6, 119.1)	Zhonghuang13 (2017-2020), Fengyuan5 (2017-2019), Hedou33 (2017-2019), Huangdou11 (2017-2018), Kedou10 (2017-2019), Meng0811 (2017-2018), Xiangfeng2 (2017-2018), Zheng1307 (2017-2019), Zhoudou28 (2017-2019), Fudou1306 (2018-2019), Hedou36 (2018-2019), Jidou30 (2018-2019), Lindou11 (2018-2019), Pudou820 (2018-2019), Shanning23 (2018-2019), Shangdou1201 (2018-2019), Shengdou4 (2018-2019), Xiangfeng4 (2018-2019), Xu0112-21 (2018-2019), Zheng1311 (2018-2019), Zhonghuang73 (2018-2019), Zhoudou33 (2018-2019), Zhudou26 (2018-2019), Andou115 (2019-2020), Handou15 (2019-2020), Hengdou6 (2019-2020), Huadou22 (2019-2020), Huangdou12 (2019-2020), Luodou1305 (2019-2020), Nannong47 (2019-2020), Pudou5110 (2019-2020), Xingnong2 (2019-2020), Weidou1897 (2019-2020), Yundou1 (2019-2020)

HHH, Huang-Huai-Hai region.

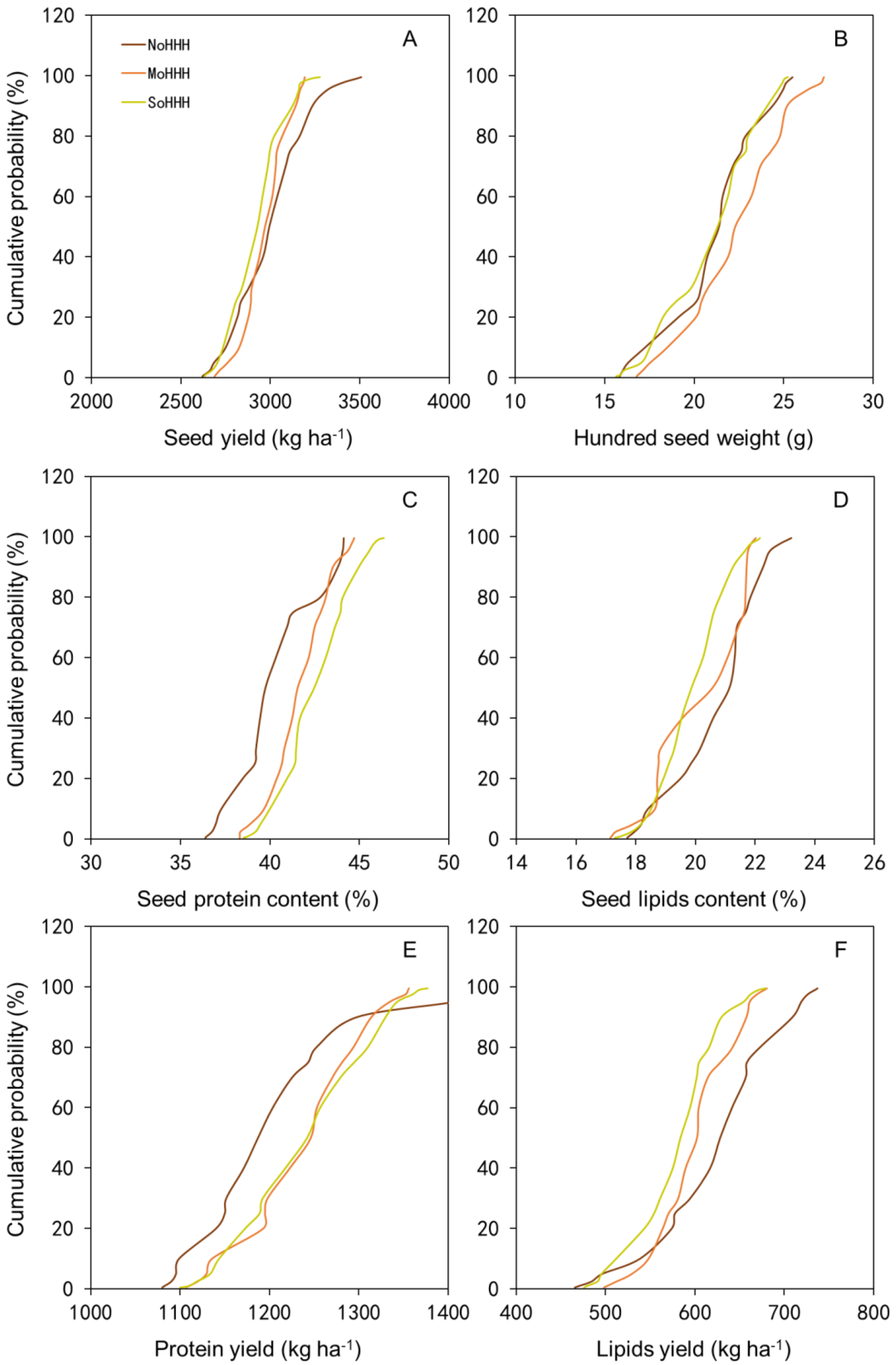

Figure S1. Cumulative probability of seed yield (A), hundred seed weight (B), seed protein content (C), seed lipids content (D), protein yield (E), and lipids yield (F) of soybeans across the Huang-Huai-Hai region, China.
